# IL-10-producing regulatory B cells induced by IL-33 (Breg^IL-33^) effectively attenuate mucosal inflammatory responses in the gut^☆^

**DOI:** 10.1016/j.jaut.2014.01.032

**Published:** 2014-05

**Authors:** Susanne Sattler, Guang-Sheng Ling, Damo Xu, Leonie Hussaarts, Andreas Romaine, Hongzhi Zhao, Liliane Fossati-Jimack, Talat Malik, H. Terence Cook, Marina Botto, Yu-Lung Lau, Hermelijn H. Smits, Foo Y. Liew, Fang-Ping Huang

**Affiliations:** a Department of Medicine, Division of Immunology & Inflammation, Imperial College, London, UK; b Department of Pathology, Li Ka Shing Faculty of Medicine, University of Hong Kong, Hong Kong, China; c Department of Paediatrics & Adolescent Medicine, Li Ka Shing Faculty of Medicine, University of Hong Kong, Hong Kong, China; d Division of Immunology, Infection and Inflammation, University of Glasgow, Glasgow, UK; e Department of Parasitology, Leiden University Medical Center, Leiden, The Netherlands; f Department of Hepatobiliary Surgery, Xinqiao Hospital, The Third Military Medical University, Chongqing, China

**Keywords:** IL-10, IL-33, Regulatory B cell, Mucosal inflammation, IBD, Breg, regulatory B cell, Breg^IL-33^, IL-33-induced/expanded Breg, IBD, inflammatory bowel disease

## Abstract

Regulatory B cells (Breg) have attracted increasing attention for their roles in maintaining peripheral tolerance. Interleukin 33 (IL-33) is a recently identified IL-1 family member, which leads a double-life with both pro- and anti-inflammatory properties. We report here that peritoneal injection of IL-33 exacerbated inflammatory bowel disease in IL-10-deficient (IL-10^−/−^) mice, whereas IL-33-treated IL-10-sufficient (wild type) mice were protected from the disease induction. A phenotypically unconventional subset(s) (CD19^+^CD25^+^CD1d^hi^IgM^hi^CD5^-^CD23^-^Tim-1^-^) of IL-10 producing Breg-like cells (Breg^IL-33^) was identified responsible for the protection. We demonstrated further that Breg^IL-33^ isolated from these mice could suppress immune effector cell expansion and functions and, upon adoptive transfer, effectively blocked the development of spontaneous colitis in IL-10^−/−^ mice. Our findings indicate an essential protective role, hence therapeutic potential, of Breg^IL-33^ against mucosal inflammatory disorders in the gut.

## Highlights

• IL-33, a branded ‘Th2’ cytokine, could accelerate the Th1-mediated colitis under an IL-10 deficient condition.
• IL-10 sufficient mice were protected from the IL-33-mediated mucosal inflammation.
• IL-33 induced a phenotypically unique subset of IL-10-producing regulatory B cells (Breg^IL-33^).
• Adoptive transfer of Breg^IL-33^ blocked the spontaneous colitis in IL-10-deficient mice (therapeutic potential).
• Our findings offer new insights into the immunological mechanisms underlying mucosal inflammation, and its regulation.

## 1 Introduction

B cells with immunosuppressive or regulatory capacity have been described for many years [1–4], although the concept of Breg as a distinct cell lineage of the immune regulatory network was not formally introduced until more recently [5,6]. Various types of Breg have since been shown including those of the B1 lineage (B1a/B10, B1b) present in the peritoneal cavity [7–9], and the transitional-2 B cells (T2-MZP, B2 lineage) found in the spleens of naïve mice [10,11]. Similar to those of regulatory T cells (Treg), their regulatory functions can be mediated by the release of immunosuppressive cytokines or molecules [5,8,11,12]. There is also evidence suggesting that Breg may serve as a tolerogenic antigen presenting cell (APC) to induce Treg, or interact directly with pathogenic T effector cells to inhibit their functions [5,13,14]. Importantly, there is now strong evidence indicating their implications in different disease conditions, including various autoimmune and inflammatory disorders both in human and animal models [6]. However, the process and factors involved in Breg induction, expansion and functions remain obscure.

Interleukin-33 (IL-33) is a recently identified IL-1-like cytokine, being the newest addition to the IL-1 super-family (IL-1F1–11) [15,16]. IL-33, also termed IL-1F11, has been classified as an “innate cytokine” associated with several inflammatory conditions, particularly of the mucosal organs and tissues [15–22]. Human IL-33 was initially detected in the airway epithelial cells, fibroblasts and smooth muscle cells constitutively, and after *in vitro* stimulation [15]. It was subsequently revealed that, in chronically inflamed tissues from patients with ulcerative colitis and Crohn's disease, endothelial cells, intestinal epithelial cells and the colonic sub-epithelial myofibroblasts (SEMFs), constituted the major source of IL-33 [17,20,23]. Abundant expression of endogenous IL-33 was found in cells of the high endothelial venues (HEV) [17] and within the inflamed mucosa [20,23]. In mice, IL-33 mRNA and protein have also been detected in various organs and tissue cells, including cells of the immune system such as macrophages and dendritic cells (DC) [15,24]. ST2, an orphan receptor of the IL-1R family cloned many years ago [25,26], and the very first cell surface lineage marker that we identified distinguishing Th2 from Th1 cells/clones [27], has recently been shown as a key component of the IL-33 receptor (IL-33R) [15]. The functional IL-33R is a heterodimer of ST2L (transmembrane form of ST2) and IL-1RAcP (IL-1R accessory protein) [28], detected on various immune cell types including Th2, B1 and mast cells [15,18,29–31].

IL-33 possesses multiple biological activities and may act in an autocrine, paracrine or even intracrine (nuclear factor) manner [15,17,32–34]. An interesting characteristic of IL-33 is that it belongs to a special subset of cytokines, including IL-1α and HMGB1 (high-mobility group box 1), which lead a “double-life” active both in their secreted and non-secreted (intracellular) forms, differentially involved in the induction and modulation of inflammatory responses [15,17,34]. The unique feature of IL-33 is however about its seemingly opposing pro-inflammatory versus anti-inflammatory properties. In contrast to its IL-1α counterpart which drives pro-inflammatory gene expression [35], the IL-33 intracellular precursor protein has been found to act instead as a co-repressor of pro-inflammatory genes, and its intra-nuclear binding could result in an anti-inflammatory phenotype [17]. It has also been shown that ST2 can dimerize with an alternative coreceptor of the IL-1R family, SIGIRR (single Ig IL-1R related molecule), acting as a negative regulator of the IL-33/ST2 signalling pathway [36]. While its ligation by IL-33 may result in the activation of MyD88, NFkB and MAPK (mitogen-activated protein kinases) in Th2 and mast cells leading to a preferential induction of Th2 cytokines (IL-5, IL-13), it may alternatively under certain condition also suppress IFN-γ (Th1) production via a hitherto unknown mechanism [15].

The immunological mechanism underlying such dichotomous functions and complex regulatory properties of IL-33 remains therefore to be understood. This may first depend on its molecular processing, release and the associated cellular compartments of the cytokine. It is now believed that, similar to the chromatin-associated nuclear factor HMGB1, the mature form of IL-33 can be released by cells undergoing necrosis. As a result of cell damage, it alerts the immune system of ‘danger’ (‘alarmins’) but otherwise remains in the nucleus of living cells where it limits immune responses [37]. IL-33 may also further exert its effects on different cell types which in return regulate each other by releasing soluble factors, as part of the immunological regulatory network or polarisation process [16].

We have recently demonstrated how necrotic cells or necrotic cell-derived factors, and their complex interactions with dendritic cells, could be directly involved in the induction of a lupus-like disease in susceptible mouse strains [38,39]. Most importantly, we showed that difference in susceptibility versus resistance to the disease induction was critically depending on the presence or absence of certain immune regulatory mechanism, and IL-10 appeared to be a crucial protective factor against the disease induction. By using IL-10 gene-knockout (IL-10^−/−^) mice, we demonstrated recently that, in the absence of IL-10 even the resistant strain could be rendered susceptible to the induction of a typical lupus-like renal disease [39].

These IL-10^−/−^ mice also develop spontaneously a Th1-mediated chronic enterocolitis, and have been widely used as an animal model of inflammatory bowel disease (IBD) [40–42]. It is well-established that the pathogenesis represents an uncontrolled Th1 response to normal gut flora due to the lack of IL-10 that normally down-regulates such a pathogenic T cell reactivity [43]. The present study was therefore designed to test and compare the effects of IL-33, a Th2 type of cytokine whose release and expression have been associated with necrotic cell death, in -systemic autoimmune and inflammatory disorders under the IL-10-deficient versus IL-10 sufficient condition. We report here that peritoneal injection of IL-33 accelerates IBD development in IL-10^−/−^ mice, but similarly treated WT mice were protected from the IL-33-mediated mucosal inflammation. More importantly, we show further how a closely associated and phenotypically unconventional subset or subsets of IL-10-producing regulatory B cells (Breg^IL-33^) induced by IL-33 may counteract against the disease induction in WT mice. These Breg^IL-33^ cells, when isolated and adoptively transferred into IL-10^−/−^ mice, can also significantly delay the onset of spontaneous IBD by restoring effectively immune homoeostasis in the IL-10 deficient recipients.

## 2 Results

### 2.1 IL-33 exacerbates enterocolitis in IL-10^−/−^ mice, whereas WT mice are protected from the IL-33-mediated mucosal inflammation

To test and compare the effects of IL-33 *in vivo* under IL-10-deficient versus IL-10-sufficient conditions, IL-10^−/−^ and WT C57BL/6 mice were injected i.p. with IL-33. The mice were then monitored for their general and clinical conditions related to IBD development, including the onset and severity of diarrhoea, rectal prolapse as well as bleeding. Blood samples were collected at regular intervals for phenotypic and functional analysis of leukocytes in circulation, and for measuring serum cytokine levels.

**Fig. 1:**
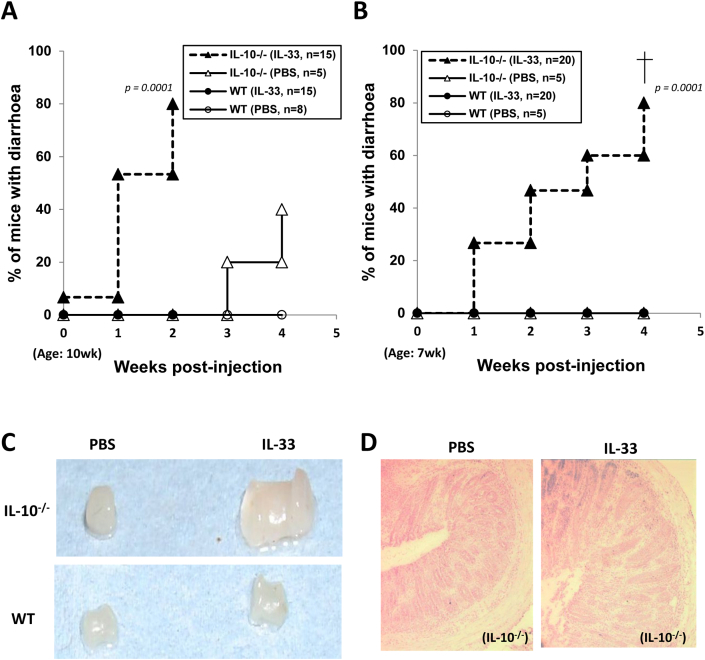

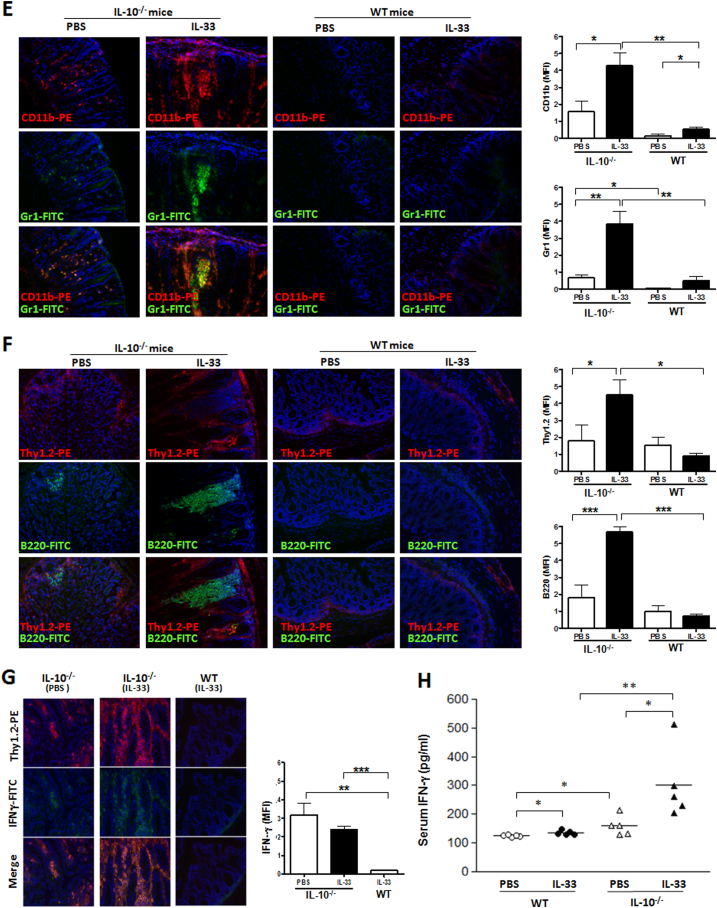
Intra-peritoneal injection of IL-33 exacerbated enterocolitis in IL-10^−/−^ mice, but similarly treated WT control mice were protected from the IL-33-mediated mucosal inflammation. Groups of IL-10^−/−^ and WT mice were injected i.p. with rhIL-33 (1 μg/mouse) twice a week, for 2 weeks starting at the age of 10 wk (A); or for 4 (B) or 6 (C–H) weeks starting from the age of 7 wk. Clinical disease activities (diarrhoea, rectal prolapse) were monitored throughout and, at the end of experiments, pathological changes in the colon and other organs or tissues assessed. A–B. Frequency of mice with signs of diarrhoea after IL-33 injection; C–F. Selected representative pictures showing colon enlargement (C), colonic histo-pathological changes (disruption of mucosal architecture, ulceration, epithelial hyperplasia) (D), and heavy leukocyte infiltration including monocytes (CD11b^+^) and neutrophils (Gr1^hi^) (E), T (Thy1.2^+^) and B (B220^+^) cells (F) as well as IFN-γ positive infiltrating T cells (G), in the colons of IL-33-injected IL-10^−/−^, but not WT control, mice. Data shown in A and B are pooled from 3 repeated experiments and the group sizes are indicated in the graphs (A, B). For further quantitative analysis of the immunostaining results (E–G, histograms), an ImageJ (NIH) picture analysis software was used to calculate mean pixel fluorescence intensity (see Methods). In order to control for background fluorescence, MFI was normalized for maximum pixel intensity. Quantitative and statistical analysis of the colonic leukocyte infiltrations was also carried out and shown (histograms) as part of E, F and G respectively (*n* = 5). H. Serum IFN-γ levels of the mice. Statistical analysis: *p* values (A–B. *Log rank test*; E–H. *Student's t test*).

IL-33 treatment accelerated enterocolitis in the IL-10^−/−^ mice, but similarly treated WT C57BL/6 mice were essentially protected from the IL-33-mediated mucosal inflammatory disease (Fig. 1). The development of spontaneous colitis in IL-10^−/−^ mice is known to be strongly influenced by environmental conditions. These mice were housed in the individual ventilated cages (IVC) as required by the IC animal facility, and kept at SPF condition with sterilized food and water. Therefore, few of them spontaneously developed diarrhoea before the age of 3 months. However, following IL-33 injection, we observed that the IL-10^−/−^ mice developed an early onset of diarrhoea. Fig. 1 shows results from two separate repeated experiments in which the mice were injected with IL-33, twice a week for two weeks starting at the age of 10-wk (A) or 7-wk (B–H) respectively. One week after IL-33 injection, some of these mice as young as 8-wks of age (Fig. 1B) started developing diarrhoea, and 80% of them had diarrhoea at the age of 11–12 weeks (Fig. 1A, B). These mice also showed signs of severe rectal prolapse observable at much younger age and some even with bleeding, as compared to the age-sex-matched PBS-treated control groups.

We had then performed detailed assessments on colonic tissues taken from the mice after different treatment schemes mentioned above (detailed in Fig. 1 legend) with various pathological changes observed. The histo- and immune-pathological data shown in Fig. 1C–H were selected from one of the experiments, in which the mice had received 12 injections starting at the age of 7 wk for 6 weeks. Fig. 1C shows representative pictures of colons collected at the end of experiments from each of the IL-33 or PBS-injected IL-10^−/−^ and WT mouse groups. The IL-33-treated IL-10^−/−^, but not WT, mice had enlarged colons (Fig. 1C, Suppl. Fig. 1A). Subsequent histological examination of the distal colon revealed typical pathological changes of IBD, featuring severe disruption of mucosal architecture, ulceration and prominent epithelial hyperplasia, in the IL-33-treated IL-10^−/−^ mice (Fig. 1D). Immunostaining of the tissue sections also revealed heavy leukocyte infiltrations, including high frequencies of monocytes (CD11b^+^Gr1^low^) and neutrophils (Gr1^hi^) (Fig. 1E), as well as T (Thy1.2^+^) and B (B220^+^) lymphocytes (Fig. 1F), in the inflamed colons. In comparison, some scattered but significantly less infiltrating leukocytes were found in the PBS-treated IL-10^−/−^ mice (Fig. 1E–F). Among those colonic infiltrating leukocytes, there was a high frequency of IFN-γ expressing T lymphocytes readily detectable in the IL-33-treated IL-10^−/−^ mice (Fig. 1G), which also correlated with serum IFN-γ levels in the mice (Fig. 1H). These results indicate that, in the absence of IL-10, IL-33 may instead enhance the pathological Th1 response.

In contrast, none of the IL-33 or PBS-treated WT control mice showed any sign of diarrhoea, rectal prolapse or bleeding, or evidence of colonic histopathological and inflammatory changes (Fig. 1E–G, and data not shown). In spite of the lack of colonic inflammation in the WT groups (Fig. 1), however, splenomegaly was clearly evident in both IL-10^−/−^ and WT mice after IL-33 treatment (Suppl. Fig. 1B). This suggested that an ongoing process of immune regulation could have occurred in the WT mice in response to IL-33.

### 2.2 IL-33 induces a subset of IL-10-producing B cells in WT C57BL/6 mice

**Fig. 2:**
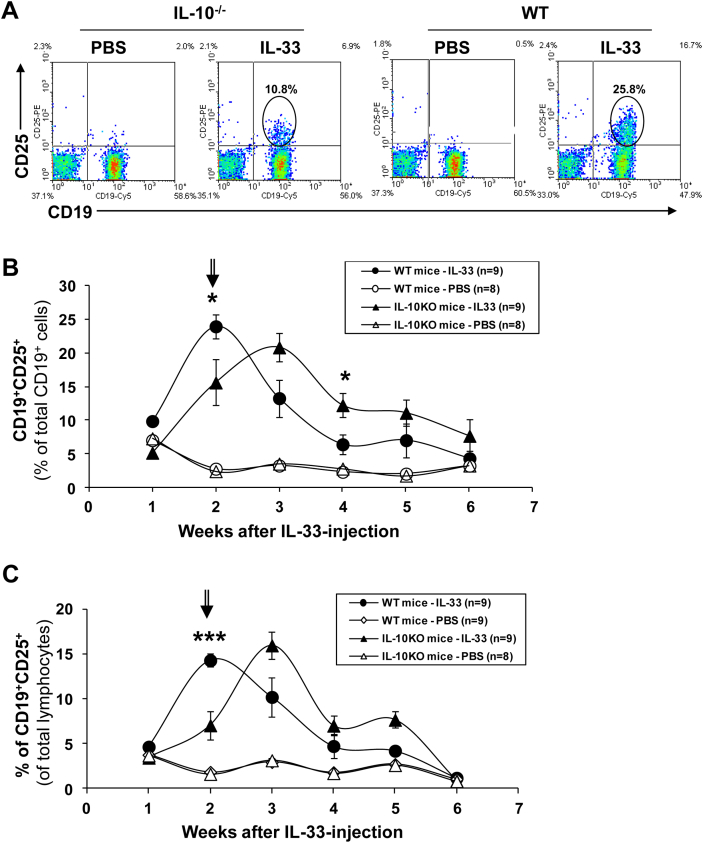
High frequencies of CD25 expressing B cells (CD19^+^CD25^+^) were detected in blood circulation of the IL-33-treated mice. Groups of IL-10^−/−^ and WT mice were injected i.p. with rhIL-33 (1 μg/mouse) twice a week, for 6 weeks starting at the age of 7 wk. The mice were bled weekly and the peripheral blood mononuclear cells (PBMC) analysed by flow cytometry using fluorescent conjugated antibodies specific for mouse CD19 and CD25. A. Selected representative FACS profiles of samples analysed at Week-2 after the 1st IL-33 injection. Kinetic changes in the frequency of CD19^+^CD25^+^ cells among total B cells (B) and of total lymphocyte population (C) were analysed. Data shown in B and C were pooled from 2 repeated experiments (*n* = 8–9). *p < 0.05; ***p < 0.001 (IL-33-treated WT versus IL-10^−/−^ groups, *Student's t test, 2 tailed*).

**Fig. 3:**
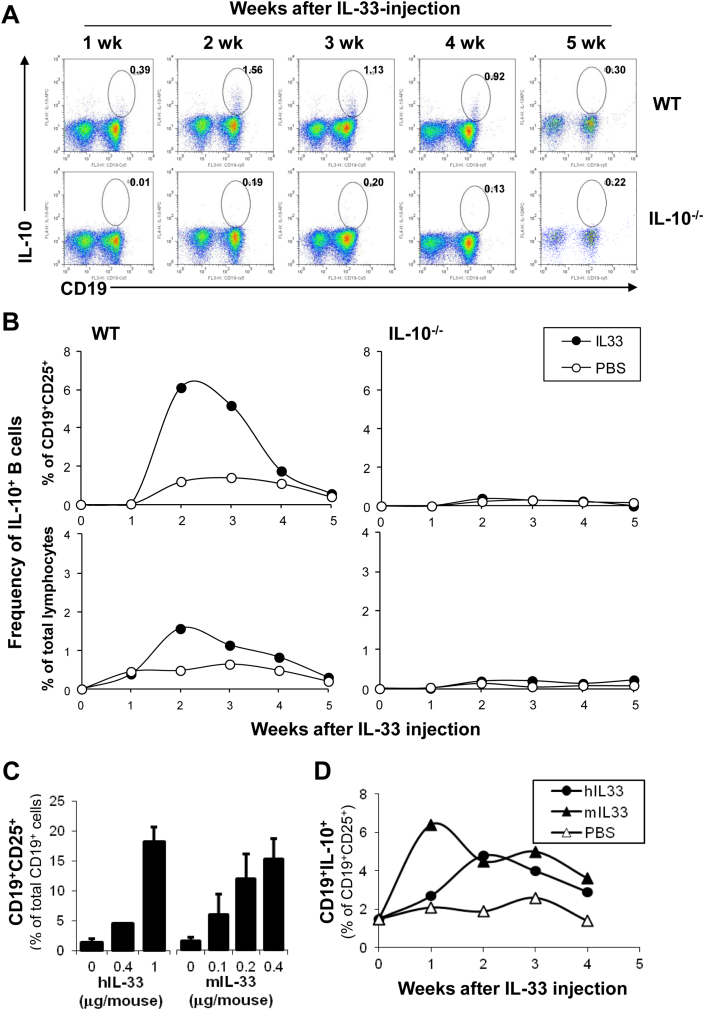
IL-33 induced a subset of IL-10 producing precursor B cells in the blood circulation of WT mice. Peripheral blood cells isolated from WT and IL-10^−/−^ mice at different time points (as indicated in A, B and D; or at 2-weeks in C) after injection with different doses (as indicated, C) or a fixed dose of recombinant hIL-33 (1 μg/mouse, A–B), mIL-33 (0.4 μg/mouse, D), or with PBS only, by procedure described in Fig. 2 legend. The cells were cultured in the presence of PMA/Ionomycin and GolgiStop for 5 h s, and analysed for intracellular IL-10 expression by FACS. A. Selected representative FACS profiles showing intracellular IL-10 expression in B cells (CD19^+^) isolated from the hIL-33 treated WT C57BL/6 mice (top panels), in comparison with those of similarly treated IL-10^−/−^ control mice (bottom panels). B. Kinetic changes in the frequency of IL-10 producing CD25^+^ B cells in blood circulation following IL-33 injection. Data shown are mean values calculated from individual mice of each group (*n* = 4) of one representative experiment. The experiments had been repeated for more than 3 times with consistent results. C & D. Direct comparison of the *in vivo* effects, potencies and time kinetics of the human versus the mouse IL-33 on B cell activation and IL-10 production. C. Dose-dependent changes in the frequency of CD25-expressing B cells appeared in blood circulation of WT mice following injection of hIL-33 or mIL33 of different dosages as indicated. Data shown are means and standard errors calculated from 3 to 5 mice per group at 2-weeks after the first IL-33 injection. D. Kinetic changes in the frequency of IL-10 producing B cells in blood circulation following injection of hIL-33 (1 μg/inj.) or mIL-33 (0.4 μg/inj.). Values were derived from staining blood samples pooled in equal volumes from 5 individual mice of each group (*n* = 5), and the experiment has been repeated for at least 5 times with consistent results.

We next investigated the immunological mechanisms underlying susceptibility versus resistance to the IL-33-mediated mucosal inflammation and, its regulation. We first analysed T cells in blood, including not only those of effector or activated phenotype but also Treg known to play an important role in intestinal homoeostasis [44]. To our surprise, no significant change in the frequency of CD25^+^ T or Foxp3^+^ T cells in the IL-33-treated mice, particularly at the early phase (Suppl. Fig. 1D, and data not shown). In contrast, however, high frequencies of CD25 expressing B cells (CD19^+^CD25^+^) were consistently detected in the blood of IL-33-injected mice of both strains, as early as one week after IL-33 injection (Fig. 2). More interestingly, these CD25^+^ B cells appeared even earlier, and with higher frequency either among total B (Fig. 2B) or total lymphocyte (Fig. 2C) population, in the WT compared to IL-10^−/−^ mice following IL-33 injection. Among them, we have subsequently identified a subset of IL-10-producing B cells, which appeared early and time-dependently in the blood of IL-33-treated WT mice (Fig. 3A–C). The induction of Breg-like cells was initially observed using human recombinant IL-33 (hIL-33) but subsequently also confirmed by using the murine IL-33 (mIL-33) when it became commercially available. The data shown in Fig. 3C and D compare their *in vivo* effects, in terms of relative potency and time kinetics, on the Breg-induction. Similar subsets of CD25^+^ and CD25^+^IL-10^+^ B cells, and some T cells at a later time point (wk-5), were also detected in the spleen and mesenteric lymph nodes (Suppl. Fig. 1C, and data not shown) though of much lower frequencies due to the heterogeneity and expansion of other cell types, of both myeloid and lymphoid lineages, in these organs. These results suggest that IL-33 could have induced a subset of IL-10-producing Breg-like cells (Breg^IL-33^) *in vivo*, protecting the WT mice from IL-33-mediated mucosal inflammation.

### 2.3 Phenotypic and functional characterization of the Breg^IL-33^

**Fig. 4:**
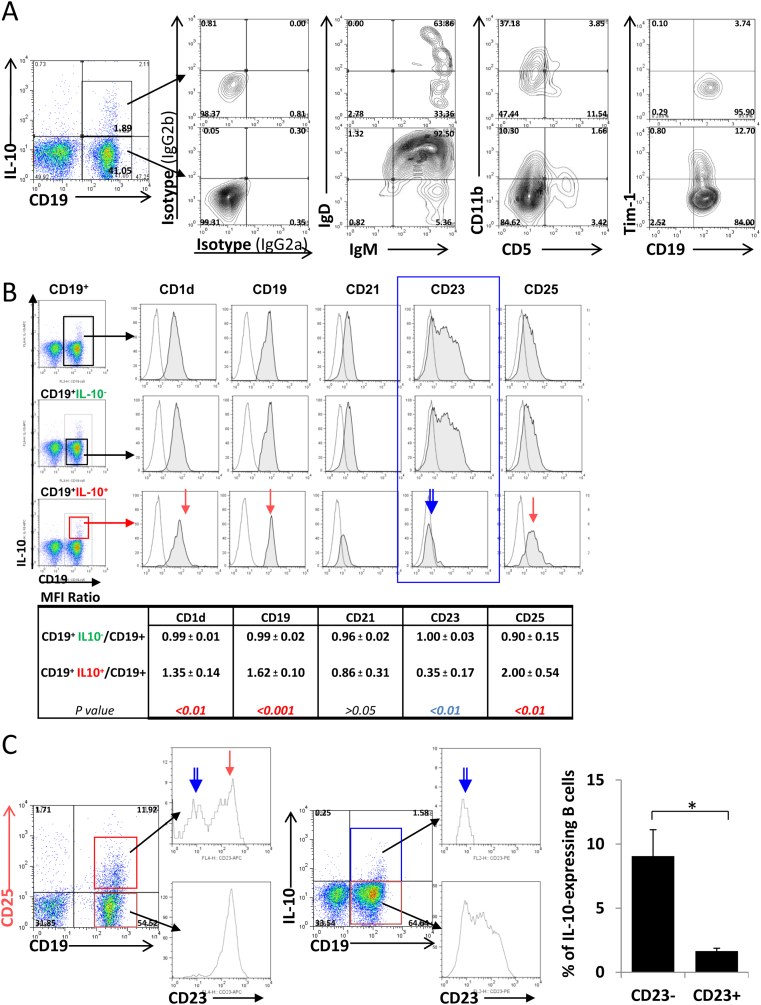

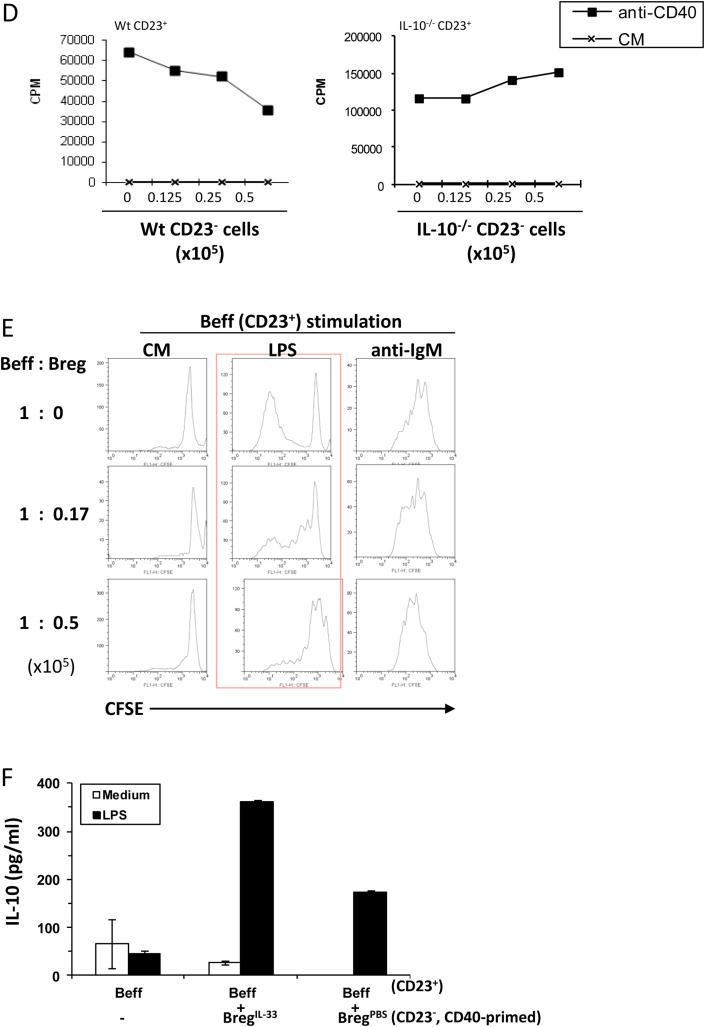
Phenotypic and functional characterization of the IL-33-induced Breg-like cells (Breg^IL-33^) in the blood. A–C. Surface phenotype of Breg^IL-33^: The IL-33-induced IL-10 producing B cells expressed high surface IgM (A), CD1d and CD25 (B), but down-regulated CD23 (FceRII) (B, C). Data shown in the table under (B) were results showing the Mean MFI (±SD) values calculated from results of 5 repeated experiments, as ratio of the IL-10^−^B, and IL-10^+^B, cell subset over the total B cells gated. Statistical analysis: *p* values (Student *t* test). D–F. *In vitro* Breg suppression assays: Immunosuppressive effects of the IL-33-induced Breg-like cells on B effector (Beff) cell proliferation (D), division (E), and its IL-10 dependency (F). D. CD23^+^ B cells (Beff, 10^5^) were cultured in the presence of anti-CD40 (2.5 μg/ml), with titrating (as indicated) doses of CD23^−^ B cells (Breg^IL-33^) purified from the IL-33-injected mice. Cell proliferation was determined by thymidine incorporation at 48 h s. E. Breg^IL-33^ (CD23^-^) purified from hIL-33-injected WT mice (Wk-2) were primed with anti-CD40 (2.5 μg/ml) for 5 h before being added to CFSE-labelled CD23^+^ Beff cells. Cell division (CFSE dilution) was determined by flow cytometry at day 5 of culture in the presence or absence of LPS (0.5 μg/ml) or anti-IgM (5 μg/ml). F. IL-10 levels in the culture supernatants were quantified by ELISA (BD Biosciences).

We then performed detailed phenotypic and functional characterization of these IL-33-induced Breg-like cells. As shown in Fig. 4, the IL-33-induced IL-10 producers had an unconventional phenotype (CD19^+^CD25^+^CD1d^hi^IgM^hi^CD5^-^CD23^-^Tim-1^-^). They expressed high surface IgM and CD1d, but lack CD5 and Tim-1 expression (Fig. 4A–B). Moreover, different from their CD19^+^CD25^-^ counterparts which were all CD23^hi^, the CD19^+^CD25^+^ population appeared to contain 2 subpopulations, one CD23^+^ and the other CD23^-/low^ (Fig. 4C). The IL-10-expressing B cells in particular had a substantially down-regulated CD23 (FcεRII) expression, being almost exclusively of CD23 negative cells (Fig. 4B, C). Such a feature allowed us to enrich and separate them from majority of the non-IL-10 producers for their functional characterization *in vitro* and *in vivo*.

To study their functionality, CD23^-^ B cells purified from IL-33-injected mice (designated Breg^IL-33^) were cultured with the CD23^+^ B cell population (designated effector B, Beff) at different ratios, and in the presence or absence of either LPS, anti-mouse CD40 or IgM antibodies. Cell proliferation was determined by thymidine incorporation. As shown in Fig. 4D, addition of the WT Breg^IL-33^ (CD23^-^) dose-dependently suppressed the proliferative response of CD23^+^ cells (Beff) to anti-CD40 stimulation. The percentage of inhibition reached 55% at the high Breg to Beff ratio (1:2). In contrast, the CD23^-^ B cells purified from IL-33-treated IL-10^−/−^ mice failed to suppress the Beff response, indicating an IL-10-dependent mechanism was involved. Instead, addition of these IL-10^−/−^ CD23^-^ cells even resulted in an increase in the total thymidine uptake values in the co-cultures (Fig. 4D, right panel).

We have also found that signalling through CD40 could effectively enhance the immunosuppressive functions of Breg^IL−33^. In brief, freshly isolated CD23^-^ B cells from IL-33-injected mice (Breg^IL−33^) were pre-primed with an anti-CD40 Ab for 5 h before adding to the CFSE-labelled CD23^+^ responder cells. The effects on Beff division in response to LPS or anti-IgM stimulation were then determined by a CFSE dilution assay. As shown in Fig. 4E, the anti-CD40-primed WT Breg^IL-33^ (CD23^−^) cells effectively inhibited Beff (CD23^+^) cell proliferation and division induced by LPS and, to some extent, anti-IgM stimulation. Similar suppressive activity though weaker was also observed when CD23^-^ B cells isolated from PBS-injected control mice (Breg^PBS^) were used (data not shown). Importantly, the suppressive effect was found to be well associated with the levels of IL-10 detected in the culture supernatants (Fig. 4F). The results above thus indicate that IL-33 induces a phenotypically unconventional subset or subsets of Breg-like cells, and their IL-10-dependent immunosuppressive capacity first confirmed *in vitro*.

### 2.4 *In vivo* therapeutic effects of Breg^IL-33^ on spontaneous IBD in IL-10^−/−^ mice

**Fig. 5:**
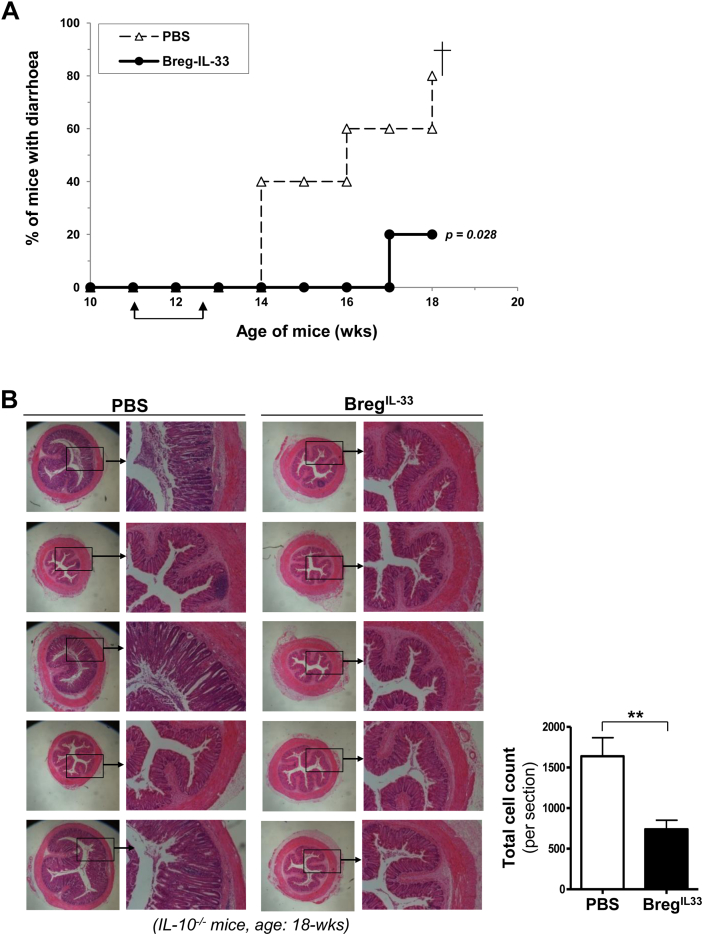
*In vivo* therapeutic effects of Breg^IL-33^ on spontaneous IBD in IL-10-deficient mice. Aged IL-10^−/−^ mice showed significantly reduced diarrhoea (A) and absence of the typical colonic histopathological changes (B), following adoptive transfer of Breg^IL-33^ (CD19^+^CD23^−^) purified from blood samples of the hIL-33-injected WT mice (Wk-2). The mice (5 per group) were injected i.v. with Breg^IL-33^ (10^6^ cells per mouse) or PBS only (control), for 2 times at the age of 11-wk and 13-wk (arrows). The treated mice were then monitored for the development of diarrhoea (A), sacrificed at the age of 18-wk (7 weeks after the 1st Breg^IL-33^ treatment), and the colons processed for histological and pathological examinations (B). For quantitative analysis (B, histograms), the ImageJ picture analysis software was used to count cell numbers by setting a signal strength threshold to select only dark nuclei and counting the number of selected particles (see Methods). The experiment has been repeated for two times with consistent results. Statistical analysis: *p values* (*MHL Log rank test*).

We next investigated the role of Breg^IL-33^ *in vivo*. CD23^-^ B cells isolated from IL-33-treated WT mice were adoptively transferred intravenously into IL-10^−/−^ mice for two times at the age of 11-wk and 13-wk. As shown in Fig. 5, the Breg^IL-33^-treated mice had improved clinical condition with a clearly reduced disease activity, compared to the PBS-treated control mice. There was a significant delay in the onset of IBD in the Breg^IL-33^-treated group, as indicated by the percentage of mice with diarrhoea (Fig. 5A). The therapeutic effects were also confirmed by histological examination of the colons. As shown in Fig. 5B, in contrast to the PBS-treated control mice which showed typical though variable pathological changes of colitis, including mucosal thickening, crypt elongation, inflammatory cell infiltration as well as crypt abscesses, no or minimal such changes were observed in all of the five Breg^IL-33^-treated IL-10^−/−^ mice. Although no significant change was found in terms of body weight (Suppl. Fig. 3A), the effects of Breg^IL-33^ treatment on splenomegaly and lymphadenopathy were also observed (Suppl. Fig. 3C–D, and explained below).

**Fig. 6:**
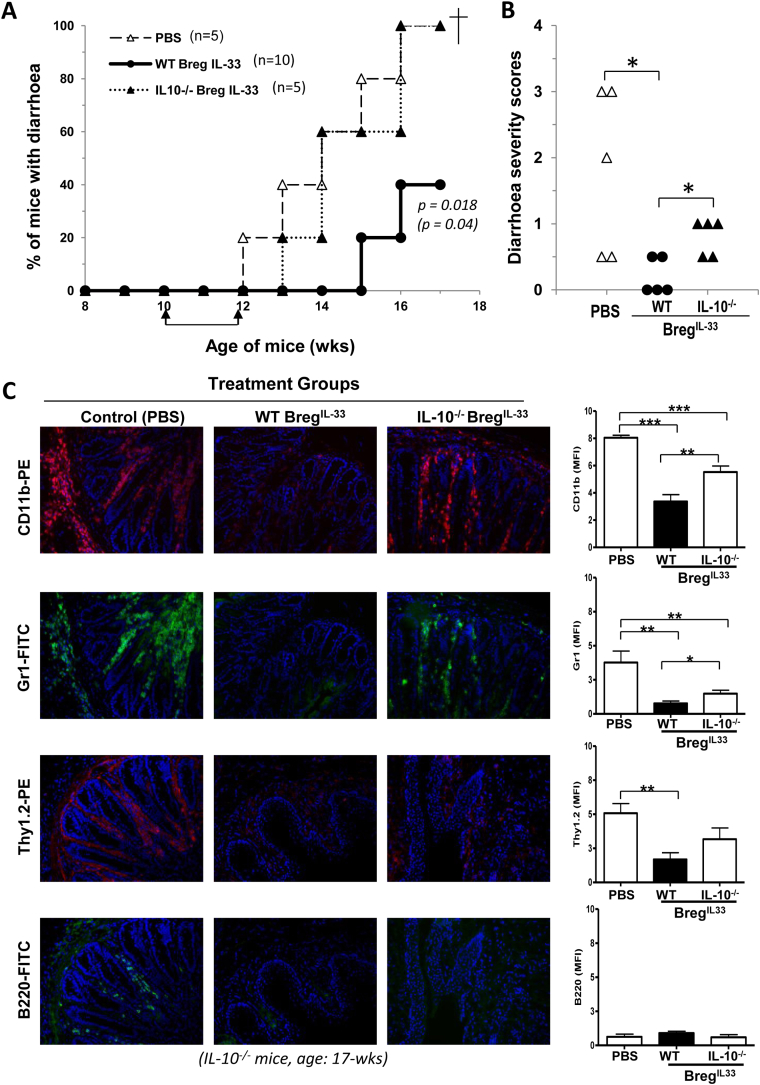

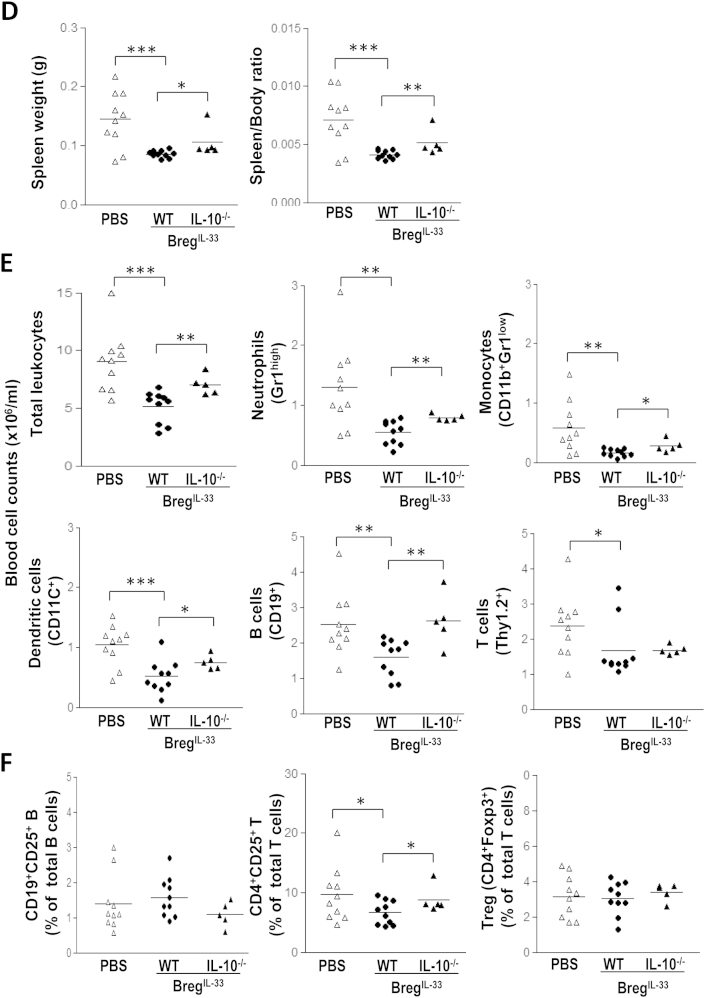

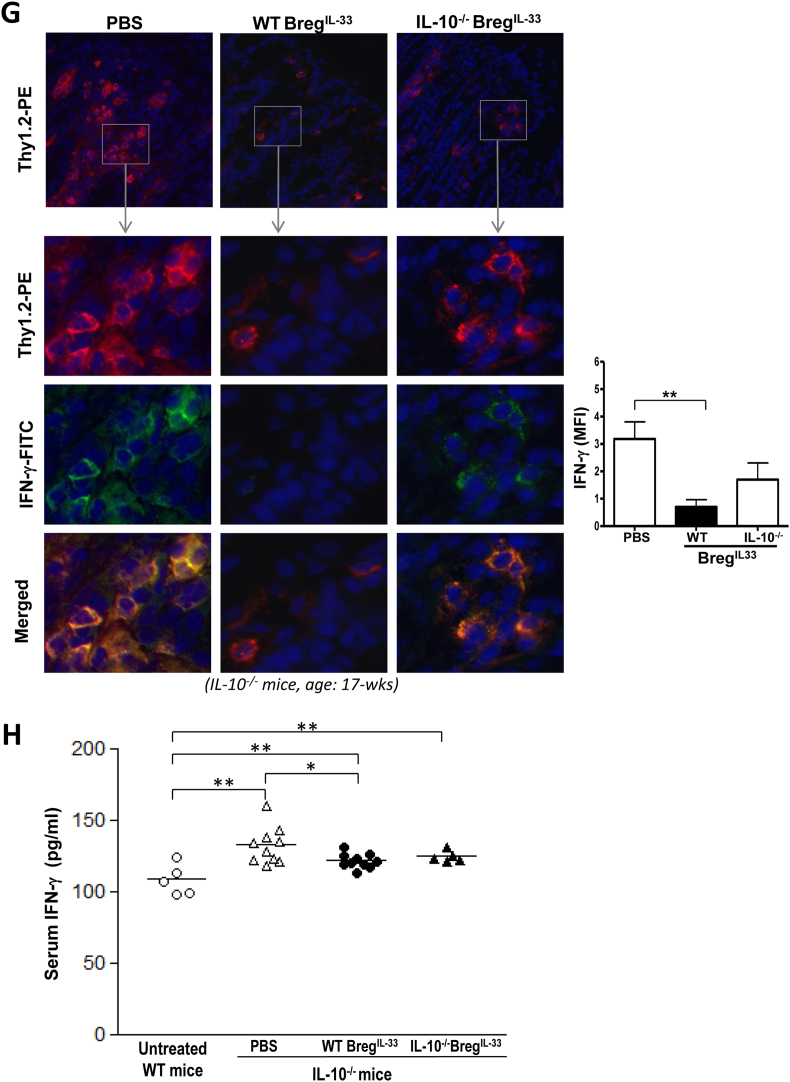
An essential role of IL-10 in the immunological mechanisms underlying Breg^IL-33^–mediated protection against spontaneous IBD in the mouse model. As described in Fig. 5 legend, groups of IL-10^−/−^ mice (5 and 10 mice per group as indicated in the graph) but at 10-wk of age were injected i.v. twice (at 10-wk and 12-wk) with CD19^+^CD23^−^ cells (10^6^ cells per mouse) purified from blood samples of the IL-33-treated WT (WT Breg^IL-33^), or IL-10^−/−^ (IL-10^−/−^Breg^IL-33^), mice. Control group was IL-10^−/−^ mice injected with PBS only (PBS). The mice were monitored twice weekly for signs of diarrhoea/disease development: A. Percentage of mice with diarrhoea. B. Diarrhoea severity scores. At the end of the experiment (7 weeks after the 1st injection, 17-wk of age), the mice were sacrificed and different organs or tissues (colon, spleen, lymph nodes) and blood samples collected and processed for assessments: C–H. Immunosuppressive effects of WT Breg^IL-33^ and IL-10^−/−^Breg^IL-33^ treatment on colonic leukocyte infiltration (C), splenomegaly (D), frequencies of different leukocytes in peripheral blood circulation (E, F) and colonic infiltrating IFN-γ expressing T cells (G), and on serum IFN-γ levels (H). For further quantitative analysis of the immunostaining results (C & G, histograms), the ImageJ (NIH) picture analysis software was used to calculate mean pixel fluorescence intensity (see Methods). In order to control for background fluorescence, MFI was normalised for maximum pixel intensity. The data shown in D–F and H are results pooled from 2 experiments and presented as individual mice (dot plots) within each of the experimental groups. Statistical analysis: A. *p* value in bold: WT Breg^IL-33^-treated group versus PBS-treated control group; *p* value in bracket: WT Breg^IL-33^-treated group versus IL-10^−/−^Breg^IL-33^-treated group (*Log rank test*). B, D–F and H: *p < 0.05, **p < 0.01, ***p < 0.001 (*Student's t test, unpaired, one-resp. two-tailed*).

To assess further the IL-10-dependency of the Breg^IL-33^ therapy, we analysed the suppressive functions of CD23^-^ B cells isolated from IL-33-treated IL-10^−/−^ mice (designated IL-10^−/−^Breg^IL-33^). Mice which received WT Breg^IL-33^ but not IL-10^−/−^Breg^IL-33^ had a significant delay in the onset of clinical disease (Fig. 6A), and a reduction in the severity (Fig. 6B), as compared to the PBS-treated mice. As shown in Fig. 6C, heavy colonic infiltration of CD11b^+^ (pan-myeloid), Gr1^high^ (neutrophils), Thy1.2^+^ (T) and B220^+^ (B) cells were evident in the PBS-treated, and the IL-10^−/−^Breg^IL-33^-treated groups. In a clear contrast, very few of these cells could be detected in the WT Breg^IL-33^-treated group. Interestingly, there was certain reduction in the colonic leukocyte infiltration observable in the IL-10^−/−^Breg^IL-33^-treated mice too (Fig. 6C, right panels). This suggests that certain IL-10-independent mechanism could also be involved in the protection, although the difference in the effects on T cells particularly Th1 cells did not reach a statistical significance based on the quantitative assessment (Fig. 6C & G histograms).

The Breg^IL-33^-mediated protection is not only evident in suppressing local inflammation in the colon, but also has a limiting effect on systemic inflammatory responses. The IL-10^−/−^ mice treated with exogenous WT Breg^IL-33^, and to a less extent IL-10^−/−^Breg^IL-33^, were clearly prevented from developing splenomegaly, a clinical condition usually caused by the inflammatory responses concomitant with IBD development. The WT Breg^IL-33^-treated group in particular had a significantly reduced spleen size (weight), with a lower spleen/body ratio, as compared to the control groups (Fig. 6D, Suppl. Fig. 3C). Similar suppressive effects were also observed on the number of leukocytes, including neutrophils (Gr1^high^), monocytes (CD11b^+^Gr1^low^), DC (CD11c^+^), B (CD19^+^) and T (Th1.2^+^) cells (Fig. 6E–F, Suppl. Fig. 3B). The neutrophils, in particular, appeared to be strongly and early responsive to the Breg^IL-33^-mediated suppression in terms of their total cell number (Fig. 6E) and ratio (time kinetics, Suppl. Fig. 3B). Among the lymphocytic populations, total B and T cell counts were also significantly decreased in the WT Breg^IL-33^ treated mice (Fig. 6E). A reduction in the frequency of CD4^+^CD25^+^ (activated) T cells was evident in the WT Breg^IL-33^-treated group, but the ratio of CD4^+^Foxp3^+^ Treg cells remained unchanged (Fig. 6F). Furthermore, the Breg^IL-33^ treatment appears to have effectively suppressed the pathogenic Th1 responses in the mice (Fig. 6G–H). While IFN-γ expression could be still readily detected among the remaining colonic infiltrating T cells in the IL-10^−/−^Breg^IL-33^-treated group, few such Th1 cells were found in the WT Breg^IL-33^-treated mice (Fig. 6G), indicating an IL-10-dependent Th1-limiting effect *in vivo*. This also correlates well with serum IFN-γ (Fig. 6H), and though less so IL-17 (Suppl. Fig. 3E), levels in the mice. Taken together, the above findings have provided a good immunological basis for the Breg^IL-33^-mediated protection against mucosal inflammatory disorders in the gut.

## 3 Discussion

In this study, we present evidence that IL-33 can be involved not only in the exacerbation but also regulation of mucosal inflammation in the gut, depending critically on the absence or presence of IL-10. We showed that IL-33, a ‘Th2’ type of cytokine, contributed significantly to IBD development in mice lacking IL-10. Similarly treated IL-10 sufficient mice were however protected from the IL-33-mediated mucosal inflammation, and the protection was closely associated with the induction of a phenotypically unconventional subset or subsets of IL-10-producing B cells. We demonstrated further, both *in vitro* and *in vivo*, how these Breg^IL-33^ cells could be responsible in protecting the WT mice from disease induction. More importantly, upon adoptive transfer into IL-10^−/−^ mice, the Breg^IL-33^ could also block the development of spontaneous IBD by restoring effectively immune homoeostasis in the IL-10 deficient recipients.

The age-dependent IBD development in the IL-10 deficient mice is known to be much affected by environmental factors including infections. However, we showed that the disease activity could be enhanced by the injection of IL-33 alone. The outcome reflects therefore a combination of the spontaneous and the IL-33-induced disease activity in terms of its disease kinetics, which were found to be related not only to the age of mice but more importantly the frequency and length of the IL-33 injections. Several recent studies have shown that the IL-33-ST2 axis is directly involved in mucosal inflammatory disorders in the gut, but some seemingly conflicting findings have been reported [45–48]. By using two experimental mouse models of chemical induced IBD, triggered either by dextran sodium sulphate (DSS) or by 2,4,6-trinitrobenzene sulphonic acid (TNBS), Sedhom and colleagues showed that injection of IL-33 could accelerate colitis, and the disease induction could be effectively prevented by blocking the IL-33-ST2 axis either through genetic ablation of the ST2 gene or the use of neutralizing antibodies [45]. In an earlier study, Imaeda and colleagues showed that while peritoneal injection of IL-33 could accelerate the disease, the treatment also prevented the depletion of goblet cells in the DSS-induced colitis, possibly through inhibition of the Notch signalling [46]. In another study by Groβ et al., interestingly, the IL-33 treatment was found to exert contrasting effects in the DSS-induced model under acute versus chronic colitis conditions. While IL-33 injection in acute colitis led to certain aggravation of inflammation, it showed extenuating effects in chronic DSS-induced colitis due to a shift of Th1 to Th2 responses [47]. Most recent findings suggest that IL-4 could play a major role in the IL-33-ST2 mediated acute inflammation in the gut [48].

Most intriguingly, our results show that IL-33, although branded as a ‘Th2’ cytokine, appears to drive an enhanced Th1 type of response under the IL-10-deficient condition. This is evident by the highly elevated serum levels of IFN-γ and the detection of colonic infiltrating leukocytes including IFN-γ+ T cells in the IL-33-injected IL-10-deficient but not WT control mice. This is also particularly interesting, as Th1 responses have long been known to be crucially involved being causative in IBD pathogenesis [49]. One possible explanation could be that IL-10 might regulate Th1 response by altering IL-33R expression. However, we found that there were comparable levels of IL-33R (ST2L) on the cells, including various B cell subsets, isolated from the IL-10-deficient and WT control mice (data not shown). Another possible explanation is that IL-33, as an innate cytokine released by necrotic cells, could be involved in orchestrating inflammation, favouring a Th1 response at an early phase of immune reaction. As a result of negative feedback regulation, i.e. due to overt inflammation at the later phase however, excessive IL-33 release and ligation may down-regulate the Th1 response possibly through an IL-10-dependent mechanism. It might do so for example by upregulating the negative regulator of IL-33/ST2 signalling (SIGIRR), and thereby shifting the response towards a Th2 phenotype [36,50]. In the absence of IL-10, however, a failure in maintaining such a homoeostasis would allow perpetuation of the pathogenic Th1-mediated pro-inflammatory responses, responsible for the uncontrolled activation, expansion and colonic infiltration of the pro-inflammatory leukocytes (neutrophils in particular) leading to IBD development.

Regulatory T cells have been shown to play an important role in maintaining intestinal homoeostasis and the lack of Treg-mediated suppression has been associated with IBD development [51]. A recent study by Duan et al. suggests that IL-33 treatment could protect against the TNBS-induced colitis by enhancing Foxp3 expression associated with Treg functions [52]. In the present study, however, we observed little change in the frequency of either Foxp3^+^ Treg or IL-10-producing CD4^+^ T cells in the blood during the early phase of IL-33 treatment. Although there was a modest effect of IL-33 on T cell expansion in the spleen at a relatively late stage, no preferential Treg expansion was observed. In contrast, B cells appeared to respond early and vigorously to IL-33 with upregulated CD25 expression. Among them, a discrete subset of IL-10-producing Breg-like cells has been identified, counteracting against the IL-33-mediated pro-inflammatory responses, potentially responsible for the protection.

These Breg^IL-33^ cells appear to have an unconventional phenotype (CD19^+^CD25^+^CD1d^hi^IgM^hi^CD5^−^CD23^−^Tim-1^−^), and do not appear to fall into either of the conventional B1 (CD5^+^) or B2 (CD23^+^) lineage. They share some phenotypic features with various Breg subsets previously described, but differ in the expression of other surface markers, including those of the B1a or B10 (CD5^+^) [8], B2 (CD23^+^) [53] and B-Tim-1 (CD5^+^Tim-1^+^) [54] IL-10-producing B cells. Though different still in terms of their heterogeneity in CD11b expression (CD11b^+^ and CD11b^−^ subsets, data not shown), they were more akin to the B1b sub-lineage (CD1d^hi^IgM^+^CD5^−^CD11b^+^CD23^−^), an innate-like B cell subset previously shown to be induced under chronic inflammatory environment associated with high IL-1 activity [9].

Importantly, we demonstrate here that these Breg^IL-33^ cells, when isolated and adoptively transferred into IL-10^−/−^ mice, can block the development of spontaneous colitis in the IL-10-deficient recipients. The Breg^IL-33^ treatment dramatically decreased the frequency of IFN-γ-expressing T cells in the colon, with significantly reduced serum IFN-γ levels. However, there was again little change in Treg frequency in the Breg^IL-33^-treated mice, indicating the therapeutic effects of Breg^IL-33^ were likely through mechanisms other than induction or expansion of Treg. The Breg^IL-33^ cells may do so by suppressing directly immune effector cells, including the IFN-γ producers. We present clear evidence here that these Breg^IL-33^ cells, by releasing IL-10, can also suppress the expansion of various inflammatory innate immune cells, again neutrophils in particular, both in the colon and in circulation of the IL-10 deficient mice. In future studies, it would also be interesting to test the effectiveness of these Breg cells in protecting against directly the IL-33-induced/accelerated disease, in addition to the age-dependent spontaneous IBD development shown in the present study.

Taken together, our findings indicate that the IL-10-producing Breg^IL-33^ play an essential protective role against mucosal inflammation. They may do so possibly by controlling the early phase of inflammatory responses, similar to the so-called innate lymphoid cells (ILCs) with regulatory functions recently described [55]. While such regulatory mechanism appeared to be succeeded in the WT strain, it failed badly in the mice lacking IL-10. The lack of IL-10 may thus not only block Treg functions as previously documented, but also prevent the induction and functions of the Breg-like cells in response to IL-33 and possibly other inflammatory mediators. These may together time-wise contribute to the break-down of immunological homeostasis in the mice, leading to or being susceptible to the induction of various autoimmune and inflammatory disorders. The present findings have therefore provided an immunological basis for the Breg^IL-33^-mediated protection, which not only offers a good explanation for the dichotomous functions of IL-33, but also points to the potential therapeutic value of Breg^IL-33^ for treating mucosal inflammatory diseases in the future.

## 4 Materials and methods

### 4.1 IL-33 reagents

The recombinant human IL-33 (hIL-33), provided by our collaborators in Glasgow with a purity of more than 95%, had been routinely tested for its endotoxin (LPS) levels (<0.01 EU/μg) by the Limulus amebocyte lysate QCL-1000 pyrogen test (BioWhittaker). Moreover, the bioactivity and specificity of our recombinant hIL-33 were also further confirmed by its ability to induce IL-5 production using Th2 cells polarized from WT and IL-33 receptor (IL-33R) knock-out (ST2^−/−^) mice (ref.30). The recombinant mouse IL-33 (mIL-33) was obtained commercially with a purity of more than 98%, and an endotoxin level less than 1.0 EU/μg, according to the supplier (R&D Systems Inc., UK).

### 4.2 Mice

Mice of the IL-10-deficient (IL-10^−/−^) strain (B6.129P2-Il-10^tm1Cgn^/J, H-2^b^; Stock no. 002251) were purchased from the Jackson Laboratory (Bar Harbor, ME, USA), and the wild type (WT) C57BL/6 strain (H-2^b^) from the Charles River Laboratories, UK. The IL-10^−/−^ mice develop spontaneously a chronic enterocolitis, and have been used widely as an animal model of inflammatory bowel disease (IBD) [40]. The intestinal inflammatory process is characterized by focal transmural inflammation, mucosal hyperplasia and extensive infiltration with CD4^+^ and CD8^+^ T cells, macrophages, IgA^+^ plasma cells and scattered neutrophils [42]. Clinical signs of inflammation include diarrhoea, perianal ulceration, intestinal bleeding and occasional rectal prolapse. The disease severity is largely depending on the housing environment. Different from the general enterocolitis observed in IL-10^−/−^ mice from conventional conditions, mice kept under specific pathogen-free conditions exhibit less severe and only local inflammation restricted to the colon [41,42]. All mice used for the present study were maintained in individual ventilated cages, and set up as breeding pairs in the Specific Pathogen Free Animal Facilities of Imperial College London. All experiments involving live animals were carried out strictly according to the protocols under licenses (PIL70/6877) approved by the UK Home Office.

### 4.3 Mouse IL-33 treatment protocol

Groups of IL-10^−/−^ and WT mice (female, 7–10 weeks of age) were injected i.p. with recombinant human IL-33 (hIL-33, 1 μg/mouse) or murine IL-33 (mIL-33, 0.4 μg/mouse) in PBS (100 μl), or with PBS only, twice a week for up to 6 weeks. The dose of IL-33 chosen was based on initial experiments in which serial doses of hIL-33 and mIL-33 were tested and compared for their potency in disease, as well as CD25^+^ B and Breg^IL-33^ induction (Fig. 3C, and data not shown). Clinical disease activities (diarrhoea) and changes of leukocytes in peripheral blood were monitored throughout and, at the end of experiments, pathological changes in the colons, mesenteric lymph nodes and spleen assessed (detailed below).

### 4.4 Cell phenotype analysis by flow cytometry

Phenotypic analysis of cells isolated from peripheral blood, spleen and lymph nodes of mice before and after IL-33, or Breg^IL-33^, injection was performed using different combinations of fluorescent-labelled antibodies specific for mouse leukocyte surface and intracellular markers. These antibodies, including anti-Thy1.2-PE, anti-CD1d-PE, anti-CD4-APC, anti-CD5-FITC, anti-CD11b-PE, anti-CD11c-APC, anti-CD19-PercPCy5, anti-CD21-FITC, anti-CD23-PE, anti-CD25-PE, anti-IgD-PE, anti-IgM-PE, anti-TIM-FITC, anti-ST2L, anti-Gr1-FITC and anti-Foxp3-PE, were obtained commercially from BD Pharmingen (UK), with the exception of anti-Foxp3 Ab (eBioscience) and anti-ST2L (MD Biosciences). For intracellular detection of Foxp3, the cells were first stained with appropriate surface markers above, followed by the cell fixation and permeabilization procedures (as described below same for intracellular cytokine detection), before incubation with the anti-Foxp3-PE antibody.

Based on their staining patterns, different cell populations and subpopulations including T (Thy1.2^+^), B (CD19^+^), helper T (Thy1.2^+^CD4^+^), regulatory T (Thy1.2^+^CD4^+^Foxp3^+^), B1a (CD19^+^CD11b^+^CD5^+^CD1d^hi^), B1b (CD19^+^CD11b^+^CD5^-^CD1d^hi^), B2 (CD19^+^CD5^-^CD1d^+^CD23^+^IgM^hi^) and B10 (CD19^+^CD5^+^CD1d^hi^CD23^-^IgM^hi^IL-10^+^) cells, neutrophils (Gr1^high^), monocytes (CD11b^+^Gr1^low^) and dendritic cells (CD11c^+^) were defined and quantified. For the flow cytometry data, gating was based on the use of respective isotype control antibodies, and the analysis was performed using FlowJo 7.6.4 (TreeStar Inc, Ashland, OR, USA).

### 4.5 Intracellular staining for IL-10 expression

IL-10 expression of the IL-33-induced Breg-like cells was measured *ex vivo* by incubating whole blood cells diluted 1:15 in RPMI1640 cell culture medium, or purified mononuclear cells (5 × 10^6^ cells/ml), and stimulating them with PMA (50 ng/ml)/Ionomycin (500 ng/ml) for 5 h at 37 °C (5% CO2) in the presence of GolgiStop (Becton Dickinson, UK). The cells were then labelled with different cell surface markers mentioned above, before being fixed and permeabilized in the BD Cytofix/Cytoperm buffer (Becton Dickinson, UK), followed by immunostaining with anti-IL-10 antibody (BD Pharmingen). Contaminated red blood cells were then lysed, white blood cells washed and re-suspended in FACS buffer (BD Biosciences) before being loaded onto FACSCalibur for analysis as described above.

### 4.6 Breg^IL-33^ isolation

Based on their phenotypic characteristics, Breg^IL-33^ cells were enriched and isolated from peripheral blood of IL-33-injected WT and IL-10^−/−^ mice for further functional characterization both *in vitro* and *in vivo*. Briefly, the mice were sacrificed at the peak of Breg^IL-33^ appearing in the blood, i.e. week-2 after the 1st IL-33 injection (a total of 5 injections) (Fig. 3). Blood was harvested by heart puncture and diluted in PBS +5% EDTA. PBMC were first isolated by density separation using Lympholyte-Mammal medium (Cedarlane Laboratories Ltd, Canada). Total B cells (CD43^-^) were then negatively selected using the MACS CD43 microbeads (Miltenyi Biotec, Germany), followed by depletion of CD23^+^ cells using an anti-CD23-PE antibody (Clone B3B4, Becton Dickinson, UK) and anti-PE microbeads (Miltenyi Biotec, Germany). All procedures were performed according to the manufacturers' instructions. Purities obtained were: total B cells (CD19^+^) >95%; of which the negatively selected population (CD23^−^ B cells) were >80%, and the remaining positively selected (CD23^+^ B cells) >90%. The flow through obtained after both depletion steps was enriched in CD23^−^ B cells with IL-10 producing capacity (∼3 folds), majority of which were also of CD25^+^ B cells (data not shown).

### 4.7 *In vitro* Breg^IL-33^ suppression assays

The *in vitro* suppressive capacity of Breg^IL-33^ on B responder cell proliferation and division was determined by the ^3^H-thymidine uptake and CFSE dilution assays. Respectively, fixed numbers (10^5^) of B responder cells (CD19^+^CD23^+^) were cultured, in the presence or absence of anti-CD40 (2.5 μg/ml, Enzo Life Sciences, UK), anti-IgM (5 μg/ml, Thermo Scientific, UK) or LPS (0.5 μg/ml, Sigma–Aldrich, UK), with titrated doses of Breg^IL-33^ (CD19^+^CD23^−^) isolated from IL-33-treated WT or IL-10^−/−^ mice as described above. In some experiments, purified Breg^IL-33^ were first primed by the anti-CD40 antibody (2.5 μg/ml, 5 h) before adding them to the responder cells. For the cell proliferation assay, 1 μCi/well ^[3H]^thymidine (Amersham, UK) was added for the last 16 h, and the thymidine incorporation determined using a scintillation counter (PerkinElmer, MA, USA). For the CFSE dilution assay, responder cells were pre-labelled with CFSE (Molecular Probes, NY, USA), before co-culturing and stimulation, and cell division (CFSE dilution) measured by flow cytometry.

### 4.8 Cytokine quantification by ELISA

Levels of cytokines including IL-10, IL-17, IFN-γ present in the mouse serum or culture supernatants were quantified by commercial ELISA kits according to the manufacturer's instructions (eBiosciences, UK).

### 4.9 Protocol for the *in vivo* Breg^IL-33^ adoptive transfer experiments

To confirm their disease-limiting effects *in vivo*, Breg^IL-33^ isolated from the IL-33-injected WT (WT Breg^IL-33^) or IL-10^−/−^ (IL-10^−/−^Breg^IL-33^), mice were adoptively transferred by intravenous injection into groups of IL-10^−/−^ mice. The recipient mice were given a total of 2 injections of Breg^IL-33^ (10^6^ cells/injection) or PBS (Control), 2 weeks apart, starting at the age of 10–11 weeks. The mice were monitored at least twice weekly for their general clinical conditions, and signs of diarrhoea, rectal prolapse and bleeding as detailed below. Blood samples were taken weekly for leukocyte phenotyping and serum cytokine levels.

### 4.10 Clinical and pathological assessments for IBD

(1) Diarrhoea monitoring

During the IL-33 and Breg^IL-33^ treatment experiments, mice were monitored and recorded for diarrhoea twice weekly (both frequency and severity). Monitoring was performed in a blinded fashion, carried out consistently during early mornings and scored according to the following scheme: First signs of mild diarrhoea (soft faeces, wet hair) (+); and obvious diarrhoea with redness and hair-loss in anal area (++), severe redness and swelling of anal area with retractable rectal prolapsed (+++), or permanent rectal prolapsed (++++).

(2) Histology for colitis assessment

Mouse colons were dissected and snap-frozen in OCT (VWR, UK) and stored at −80 °C, or fixed in Bouin's fixative for 4 h and embedded in paraffin, for histological analysis. The tissues were cut (5 μm thick), and cryosections fixed in cold acetone for 5 min and air-dried, then stained with haematoxylin for 1 min and examined using light microscopy.

(3) Immunostaining of colon tissue sections

The mouse colon cryosections were fixed in acetone and stained with fluorescence-labelled antibodies specific for cell surface markers of B cells (anti-B220-FITC), T cells (anti-Thy1.2-PE), myeloid cells (anti-CD11b-PE) and neutrophils (anti-Gr1-FITC), and IFN-γ (anti-IFN-γ-FITC) (all obtained from Cambridge Biosciences, UK). Cell nuclei were visualized using DAPI containing mounting fluid (Dako UK Ltd, UK), and the sections were analysed using an Olympus BX4 fluorescence microscope (Olympus, UK). For further quantitative analysis of the colonic pathological changes, the ImageJ (NIH) picture analysis software [56,57] was used to calculate mean pixel fluorescence intensity and to count cell numbers. In order to control for background fluorescence, MFI was normalized for maximum pixel intensity. Cell numbers were calculated by setting a signal strength threshold to select only dark nuclei and counting the number of selected particles.

### 4.11 Statistical analysis

Mantel–Haenszel log rank (MHL) test was used to analyse the statistical significance of differences between mouse groups (diarrhoea development), in the IL-33 and Breg^IL-33^ treatment experiments. Student's *t*-test was used for pair-wise comparison between experimental groups in terms of changes in leukocyte frequencies and cytokine levels. *p* ≤ 0.05 (*), *p* ≤ 0.01 (**), *p* ≤ 0.001 (***).

## Conflict of interest

The authors declare no financial conflict of interest.
