# Supplementary material for: IL-10-producing regulatory B cells induced by IL-33 (BregIL-33) effectively attenuate mucosal inflammatory responses in the gut
Source: J Autoimmun. 2014 May;50(100):107–22. doi: 10.1016/j.jaut.2014.01.032 (PMC4012142; doi:10.1016/j.jaut.2014.01.032)
Supplement: Supplementary file 1 [file mmc1.ppt]

## Slide 1
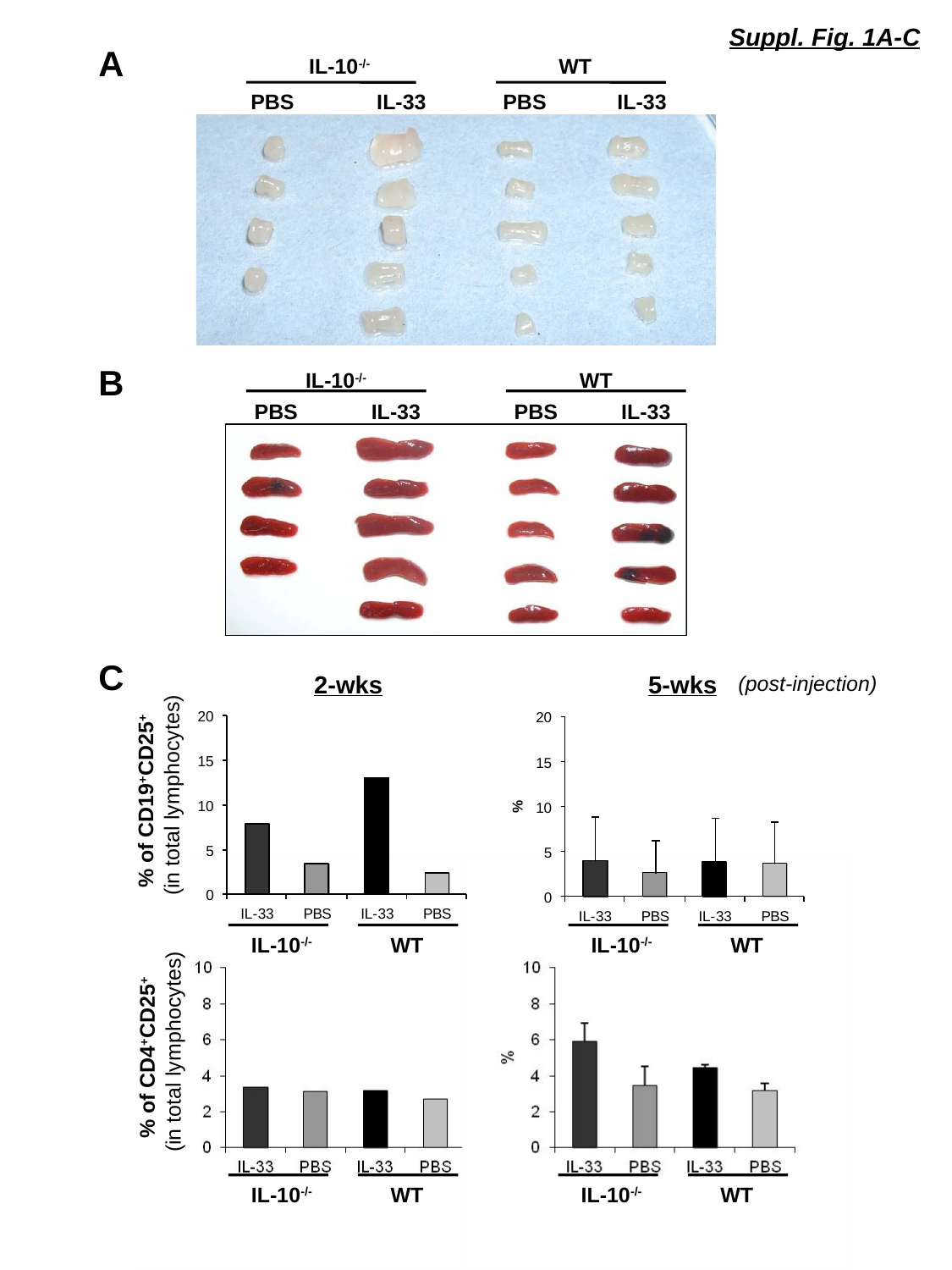

Suppl. Fig. 1A-C
A
IL-10-/- WT
PBS IL-33 PBS IL-33
B
IL-10-/-
WT
PBS
IL-33
PBS
IL-33
C
2-wks
5-wks
(post-injection)
% of CD19+CD25+
 (in total lymphocytes)
IL-10-/-
WT
IL-10-/-
WT
% of CD4+CD25+
 (in total lymphocytes)
IL-10-/-
WT
IL-10-/-
WT

## Slide 2
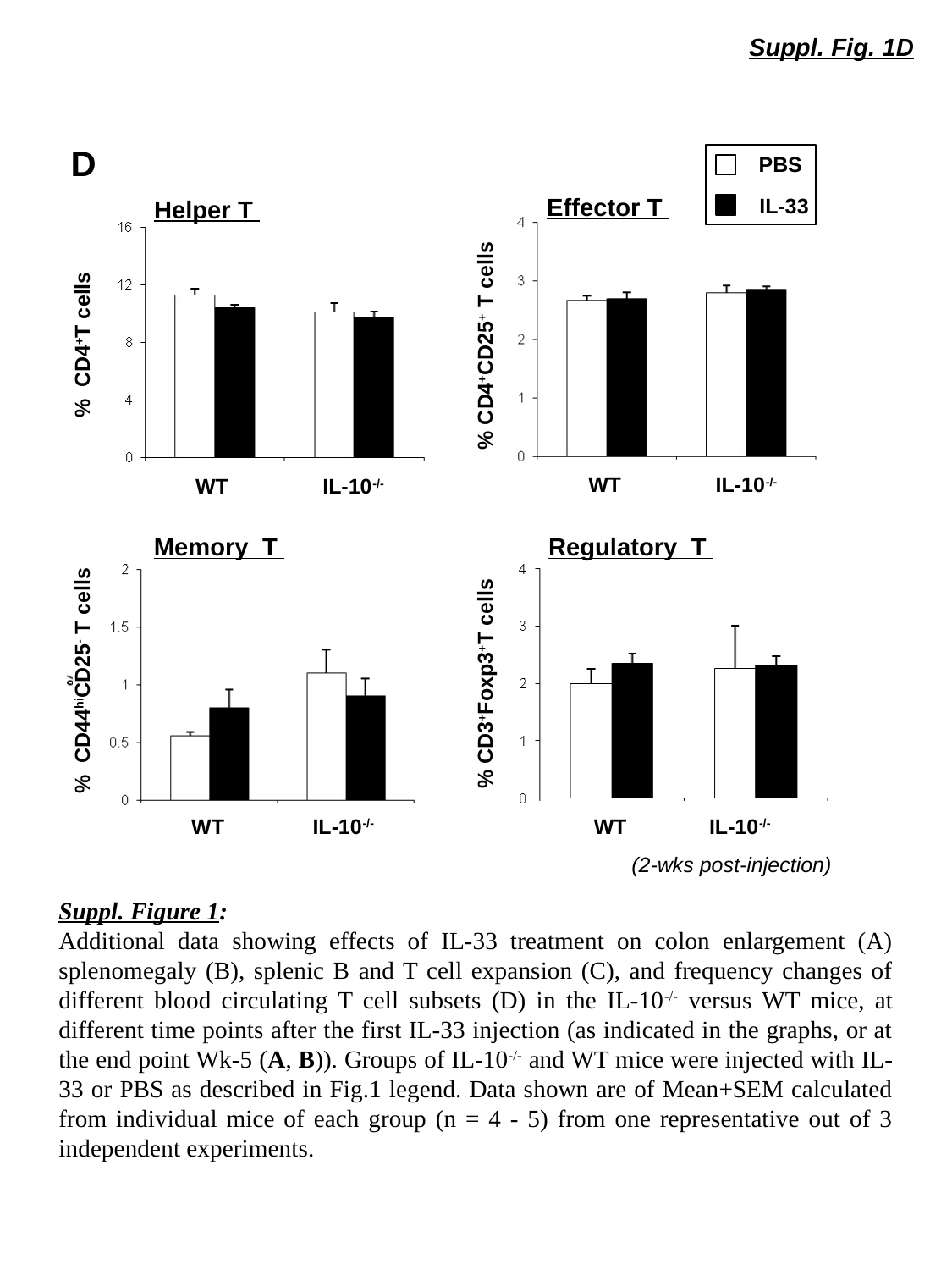

Suppl. Fig. 1D
D
PBS
Effector T
IL-33
Helper T
% CD4+T cells
% CD4+CD25+ T cells
WT IL-10-/-
WT IL-10-/-
Memory T
Regulatory T
% CD44hiCD25- T cells
% CD3+Foxp3+T cells
WT IL-10-/-
WT IL-10-/-
(2-wks post-injection)
Suppl. Figure 1:
Additional data showing effects of IL-33 treatment on colon enlargement (A) splenomegaly (B), splenic B and T cell expansion (C), and frequency changes of different blood circulating T cell subsets (D) in the IL-10-/- versus WT mice, at different time points after the first IL-33 injection (as indicated in the graphs, or at the end point Wk-5 (A, B)). Groups of IL-10-/- and WT mice were injected with IL-33 or PBS as described in Fig.1 legend. Data shown are of Mean+SEM calculated from individual mice of each group (n = 4 - 5) from one representative out of 3 independent experiments.

## Slide 3
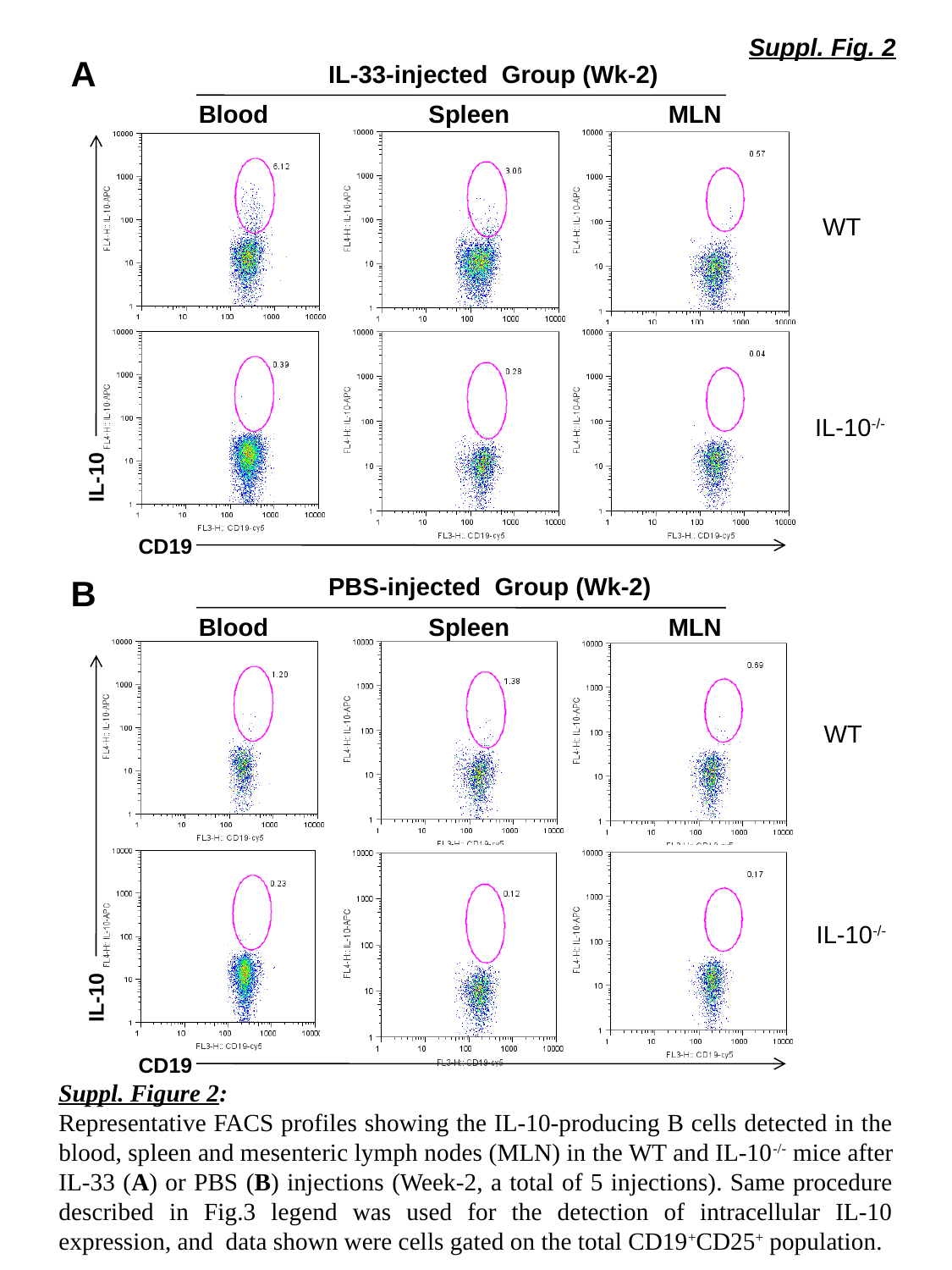

Suppl. Fig. 2
A
IL-33-injected Group (Wk-2)
Blood
Spleen
MLN
WT
IL-10-/-
IL-10
CD19
B
PBS-injected Group (Wk-2)
Blood
Spleen
MLN
IL-10
WT
IL-10-/-
IL-10
CD19
Suppl. Figure 2:
Representative FACS profiles showing the IL-10-producing B cells detected in the blood, spleen and mesenteric lymph nodes (MLN) in the WT and IL-10-/- mice after IL-33 (A) or PBS (B) injections (Week-2, a total of 5 injections). Same procedure described in Fig.3 legend was used for the detection of intracellular IL-10 expression, and data shown were cells gated on the total CD19+CD25+ population.

## Slide 4
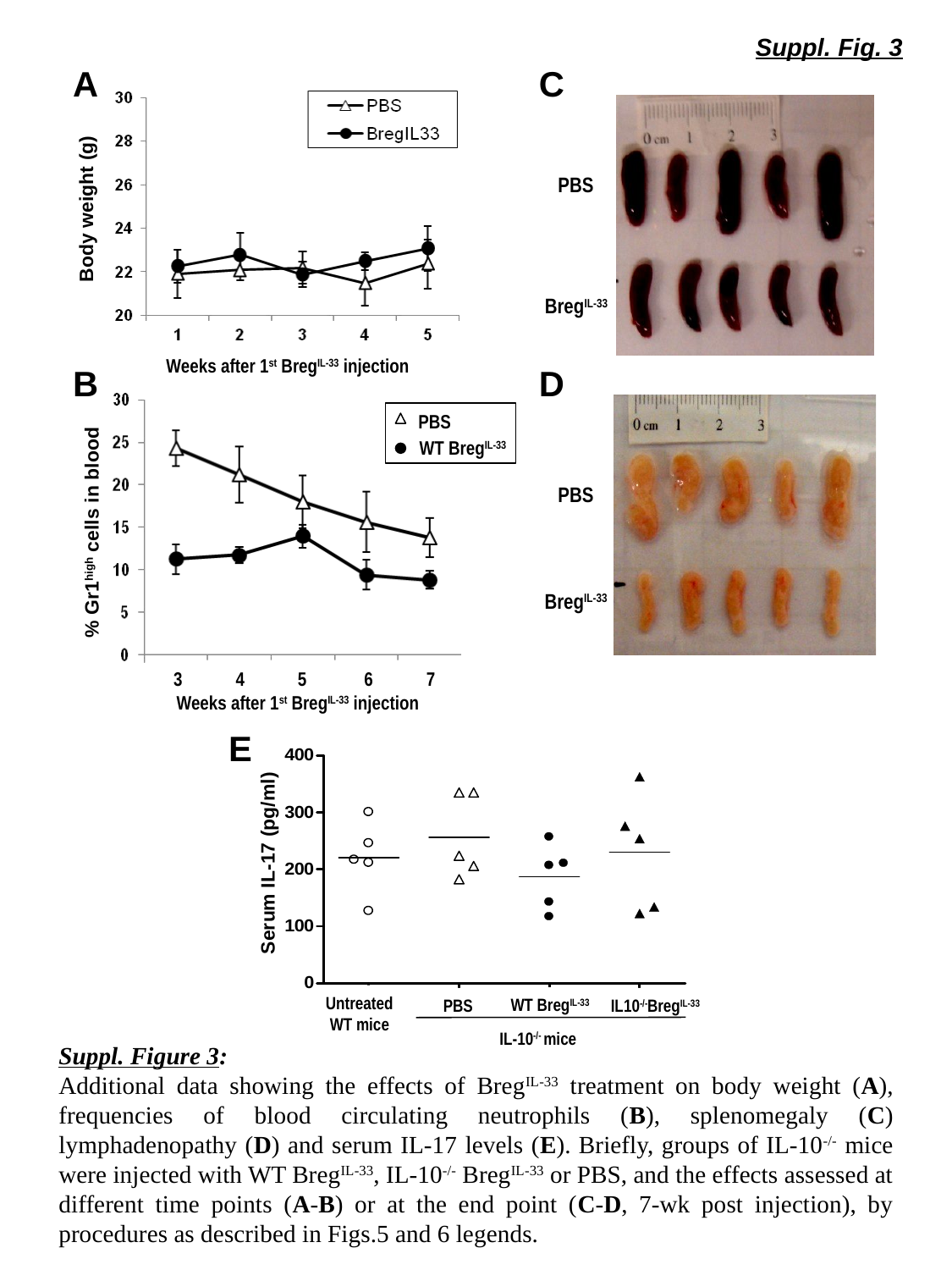

Suppl. Fig. 3
A
C
PBS
Body weight (g)
BregIL-33
Weeks after 1st BregIL-33 injection
B
D
PBS
WT BregIL-33
PBS
% Gr1high cells in blood
BregIL-33
3 4 5 6 7
Weeks after 1st BregIL-33 injection
E
Serum IL-17 (pg/ml)
Untreated
WT mice
WT BregIL-33
 PBS
IL10-/-BregIL-33
IL-10-/- mice
Suppl. Figure 3:
Additional data showing the effects of BregIL-33 treatment on body weight (A), frequencies of blood circulating neutrophils (B), splenomegaly (C) lymphadenopathy (D) and serum IL-17 levels (E). Briefly, groups of IL-10-/- mice were injected with WT BregIL-33, IL-10-/- BregIL-33 or PBS, and the effects assessed at different time points (A-B) or at the end point (C-D, 7-wk post injection), by procedures as described in Figs.5 and 6 legends.
